# The primary cilium conducts chondrocyte mechanotransduction

**DOI:** 10.1186/2046-2530-1-S1-P59

**Published:** 2012-11-16

**Authors:** AKT Wann, N Zuo, CJ Haycraft, CG Jensen, CA Poole, SR McGlashan, MM Knight

**Affiliations:** 1 Queen Mary, University of London,UK; 2 University of Auckland, New Zealand; 3 University of South Carolina, USA; 4 University of Otago, New Zealand

## 

In several cell types fluid-flow deflection of primary cilia initiates a mechanotransduction pathway via polycystin 1 and 2 (PC1/2). In articular cartilage the chondrocyte primary cilium extends into cartilage matrix. Mechanical signals including compression and fluid flow trigger mechanosensitive regulation of matrix synthesis underpinning tissue homeostasis. Here we tested the hypothesis that the cilium plays a key role in chondrocyte mechanotransduction that includes ATP release, ATP-induced calcium transients and the subsequent regulation of matrix synthesis. These studies used murine chondrocytes with a hypomorphic mutation of *Tg737*, (encoding IFT88) which abolishes cilia growth. 3D agarose culture allowed compressive loading of WT and *Tg737* chondrocytes followed by quantification of ATP release with a luciferase assay, calcium transients by Fluo-4 imaging, and matrix synthesis by qPCR and biochemical assay. Additionally, expression of purinergic receptors (P2R) and polycystins was assessed by western blot and immunocytochemistry. Compression of WT chondrocytes increased calcium transients and matrix production. By contrast this mechanosensitive behaviour was completely abolished in *Tg737* cells. However mechanosensitive ATP release was present in both WT and *Tg737* cells suggesting that IFT88 and the cilium are required for purinergic reception. Indeed exogenous ATP elicited calcium transients in WT but not in *Tg737* cells. P2R expression profiles showed no global differences but polycystin-1 expression was altered in ORPK. We conclude that IFT88 plays a critical role in ATP-induced calcium signalling and is therefore essential to chondrocyte mechanotransduction. Furthermore, this suggests that IFT88 and the cilium may be fundamentally important for purinergic-calcium signalling pathways.

